# Cancer Incidence among Pesticide Applicators Exposed to Dicamba in the Agricultural Health Study

**DOI:** 10.1289/ehp.9204

**Published:** 2006-07-13

**Authors:** Claudine Samanic, Jennifer Rusiecki, Mustafa Dosemeci, Lifang Hou, Jane A. Hoppin, Dale P. Sandler, Jay Lubin, Aaron Blair, Michael C.R. Alavanja

**Affiliations:** 1 Division of Cancer Epidemiology and Genetics, National Cancer Institute, National Institutes of Health, Department of Health and Human Services, Bethesda, Maryland, USA; 2 Uniformed Services University of the Health Sciences, Department of Preventive Medicine and Biometrics, Bethesda, Maryland, USA; 3 Epidemiology Branch, National Institute of Environmental Health Sciences, National Institutes of Health, Department of Health and Human Services, Research Triangle Park, North Carolina, USA

**Keywords:** cancer incidence, farming, neoplasms, pesticides, United States

## Abstract

**Background:**

Dicamba is an herbicide commonly applied to crops in the United States and abroad. We evaluated cancer incidence among pesticide applicators exposed to dicamba in the Agricultural Health Study, a prospective cohort of licensed pesticide applicators in North Carolina and Iowa.

**Methods:**

Detailed pesticide exposure information was obtained through a self-administered questionnaire completed from 1993 to 1997. Cancer incidence was followed through 31 December 2002 by linkage to state cancer registries. We used Poisson regression to estimate rate ratios and 95% confidence intervals for cancer subtypes by tertiles of dicamba exposure. Two dicamba exposure metrics were used: lifetime exposure days and intensity-weighted lifetime exposure days (lifetime days × intensity score).

**Results:**

A total of 41,969 applicators were included in the analysis, and 22,036 (52.5%) reported ever using dicamba. Exposure was not associated with overall cancer incidence nor were there strong associations with any specific type of cancer. When the reference group comprised low-exposed applicators, we observed a positive trend in risk between lifetime exposure days and lung cancer (*p* = 0.02), but none of the individual point estimates was significantly elevated. We also observed significant trends of increasing risk for colon cancer for both lifetime exposure days and intensity-weighted lifetime days, although these results are largely due to elevated risk at the highest exposure level. There was no apparent risk for non-Hodgkin lymphoma.

**Conclusions:**

Although associations between exposure and lung and colon cancer were observed, we did not find clear evidence for an association between dicamba exposure and cancer risk.

## Background

Dicamba (3,6-dichloro-2-methoxybenzoic acid) is a benzoic acid herbicide used to control annual and perennial broadleaf weeds in grain crops, grasslands, and non-crop areas such as fence rows and roadways. Dicamba may be used in combination with other herbicides, such as 2,4-dichlorophenoxyacetic acid (2,4-D) or atrazine. In 2001 Dicamba ranked 24th of the 25 most commonly used agricultural pesticides with 7–10 million pounds applied, and 7th of the 10 most commonly used home and garden pesticides (Kiely et al. 2004).

Dicamba was first registered for use in 1967 (National Pesticide Information Center 2005). The U.S. Environmental Protection Agency (EPA) has classified this general use pesticide as toxicity class III—slightly toxic (Extension Toxicology Network 1996), and as a Group D carcinogen: “not classifiable as to human carcinogenicity” (U.S. EPA 2004). There is no experimental evidence that dicamba is mutagenic or carcinogenic (Extension Toxicology Network 1996; U.S. EPA 2005), although rats fed dicamba at high levels over long periods experienced liver changes and decreased body weight (Edson and Sanderson 1965). Limited animal evidence suggests that dicamba could induce tumors through epigenetic mechanisms (Espandiari et al. 1998, 1999).

In epidemiologic studies, occupational use of dicamba has been associated with non-Hodgkin lymphoma (NHL; McDuffie et al. 2001) and multiple myeloma (Burmeister 1990). More recently, in the Agricultural Health Study (AHS) cohort, dicamba was associated with an increased risk of lung cancer in a nested case–control analysis (Alavanja et al. 2004). Dicamba has been found in carpet dust samples, with higher levels from states with higher prevalence of lawn and garden use (Colt et al. 2005), and in 1.4% of urine samples collected between 1976 and 1980 from 6,990 participants in the Second National Health and Nutrition Examination Survey (Kutz et al. 1992).

Despite its common use and some epidemiologic evidence that suggests increased cancer risk, existing evaluations of dicamba-related health effects are inadequate. In an effort to add to existing knowledge, as well as follow-up on the suggested association with lung cancer previously observed in this cohort, we investigated site-specific cancer incidence and risk among pesticide applicators exposed to dicamba in the AHS cohort.

## Materials and Methods

### Cohort enrollment and follow-up

The AHS is a prospective cohort study of 57,311 private and commercial pesticide applicators in Iowa and North Carolina recruited between 1993 and 1997 (AHS 2006b). Cohort members were matched to cancer registry files in Iowa and North Carolina for case identification and to state death registries and the National Death Index (Centers for Disease Conrol and Prevention 2006) to ascertain vital status. We included incident cancers diagnosed between date of enrollment and 31 December 2002. Cancers were coded according to the *International Classification of Diseases for Oncology* (ICD-O-2; New South Wales Department of Health 2000). Surviving cohort members no longer residing in Iowa or North Carolina were identified through current address records of the Internal Revenue Service, state motor vehicle registration offices, and pesticide license registries of state agricultural departments. These individuals were censored from cancer incidence follow-up on the dates they departed Iowa or North Carolina. The average period of follow-up time was 7.3 years. This study was approved by all appropriate Institutional Review Boards. Verbal informed consent was obtained from all participants.

### Exposure assessment

A self-administered enrollment questionnaire sought comprehensive information on use of 22 specific pesticides, ever/never use information for 28 additional pesticides, pesticide mixing and application methods, repair of application equipment, use of personal protective equipment (PPE), and demographic and lifestyle characteristics such as smoking status, alcohol consumption, and personal and family medical histories. This questionnaire may be accessed at the AHS website (AHS 2006a).

We used two exposure metrics for this analysis: lifetime exposure days and intensity-weighted lifetime exposure days. We estimated the number of lifetime exposure days to dicamba on the basis of the number of years applied and the frequency of application, using the midpoints of the questionnaire categories for total years of use and days per year of use. Lifetime exposure days were grouped into tertiles on the basis of the distribution among all cancer cases combined. To more finely explore potential dose–response curves, we divided the highest tertile at the median, producing the following categories for lifetime exposure days: No exposure, 1 to < 20 days, 20 to < 56 days, 56 to < 116 days, and ≥ 116 days.

To account for factors that may increase or decrease exposure, we incorporated the AHS exposure-intensity algorithm that is based on the following formula: Intensity level = [(mixing status + application method + equipment repair status) × personal protective equipment (PPE) score] (Dosemeci et al. 2002). Scores were assigned to each of the four factors that the formula comprises, which served to weight each factor according to each possible outcome defined by what was reported on the enrollment questionnaire. For example, mixing status was a three-level variable based on never mixing, personally mixing < 50% of the time, and personally mixing > 50% of the time (Mix score = 0, 3 ,9, respectively). Pesticides are typically purchased in concentrated form and must be diluted prior to application, and the diluted material is then transferred to application containers. This mixing and loading process provides opportunities for greater pesticide exposure due to contact with contaminated surfaces, splashes, and spills.

Application method was a six-level variable which, for herbicides, was based on never applying, aerial application or distribution of tablets, application in furrow, use of tractor-mounted boom sprayer, use of backpack, and use of hand spray (application score = 0, 1, 2, 3, 8, 9, respectively). The use of backpack sprayers or hand sprayers to apply pesticides may result in higher dermal exposures than use of a tractor-mounted boom sprayer because the backpack and hand spray wands are in direct contact with the applicator’s hands or body. Equipment repair status was a two-level variable based on not repairing or repairing (repair score = 0, 2, respectively). PPE was an eight-level variable based on four groups of PPE combinations (PPE = 0 if never used PPE; PPE = 1 if wore face shield/goggles or fabric/leather gloves or other protective clothing; PPE = 2 if wore cartridge respirator/gas mask or disposable outer clothing; and PPE = 3 if wore chemical resistant rubber gloves).

Intensity-weighted lifetime exposure days is the product of lifetime exposure days and intensity level, and was categorized into tertiles with the upper tertile further divided at the median. Categories included no exposure, 1 to < 86.6 intensity-weighted days, 86.6 to < 344.3 intensity-weighted days, 344.3 to < 739.2 intensity-weighted days, and ≥ 739.2 intensity-weighted days.

### Data analysis

Rate ratios (RRs) and 95% confidence intervals (CIs) were calculated to estimate risk of various cancers associated with dicamba exposure. All models were based on the Poisson distribution and included first primary incident cancer cases. Individuals with cancer prevalent at the time of enrollment (*n* = 1,075) were excluded from the analysis. Of the remaining participants, 6,362 were missing information about dicamba use and were also excluded. Because there were only four exposed cancer cases, we were unable to adequately examine risk among the 1,297 female applicators. A total of 41,969 male applicators remained in the analysis after losing 6,608 additional participants who were missing one or more covariates.

We examined risk for all cancer sites classified under ICD-O-2 (New South Wales Department of Health 2000). In the tables we present only cancers for which there were at least five cases in each exposure category. All models were adjusted for baseline variables: age at enrollment (< 40, 40–49, 50–59, ≥ 60 years), race (white, nonwhite), alcohol consumption during the year of enrollment (ever, never), smoking status at enrollment (never, low, and high based on the median value of pack-years among smokers), family history of cancer in first degree relatives (yes, no), applicator status (private, commercial), and state of residence (Iowa, North Carolina). Because of potential concurrent exposure to other pesticides, we examined the impact of adjusting for pesticides highly correlated with use of dicamba, as well as adjusting all RRs for total lifetime days of pesticide use as a continuous variable (Alavanja et al. 2004). In addition to tertiles of lifetime exposure days and intensity-weighted lifetime exposure days, we examined the association between cancer risk and tertiles of each exposure component: days per year of dicamba use, total years of dicamba use, and the dicamba intensity score. We analyzed exposure–response trends by including the midpoint of each tertile as a continuous variable in the model and testing for the statistical significance of the slope.

To evaluate the most appropriate reference group, we examined a number of characteristics that may be related to intensity of lifetime days of dicamba exposure, which was divided into three categories: those who never applied dicamba, applicators in the lowest tertile of lifetime exposure days (low-exposed), and applicators in the top two tertiles of lifetime exposure days (high-exposed). Differences with respect to these and other baseline characteristics may introduce residual confounding from a variety of unidentified sources. To avoid this, we assumed that applicators with baseline characteristics more similar to those of the applicators in the higher exposure group would be most appropriate as a reference group for the Poisson regression analyses (Rusiecki et al. 2004).

## Results

Selected characteristics of the dicamba-exposed and non-dicamba-exposed applicators are presented in Table 1. Among 41,969 participants with complete exposure information, 22,036 (52.5%) reported ever having personally applied or mixed dicamba and had complete data on lifetime days of exposure. The study population comprised primarily white, male private applicators. In both the exposed and nonexposed groups, about half of the participants reported that they had never smoked. High-exposed applicators tended to be more similar to low-exposed applicators than never-exposed applicators with respect to baseline characteristics of age, race, state of residence, smoking status, education, alcohol consumption during year of enrollment, family history of cancer, applicator type, living or working on a farm at enrollment, and corn and soybean production. Despite differences between the never-exposed and low-exposed groups, results differed only slightly when either was used as the reference group, and we provide results for both analyses.

We found no strong associations between any cancer site and dicamba exposure for either lifetime exposure days or intensity-weighted lifetime exposure days (Tables 2 and 3). We did observe a trend of increasing lung cancer risk with increasing lifetime exposure days when the referent group was low-exposed participants (*p*-value for trend = 0.02), with a RR of 2.16 (95% CI, 0.97–4.82) for the upper half of the highest tertile. The number of non-smoking, dicamba-exposed lung cancer cases (*n* = 5) was too small to assess risk among non-smokers. In an analysis based on intensity-weighted exposure days, those with exposure in the top two tertiles had nearly twice the risk as those with low exposure, but no dose response was evident. There was no evidence of increased lung cancer risk with either lifetime exposure days or intensity-weighted lifetime days when applicators unexposed to dicamba served as the referent.

We also observed significant trends of increasing risk for colon cancer for both lifetime exposure days and intensity-weighted lifetime days when the referent group comprised low-exposed applicators. However, only RRs for the highest exposure category were significant (lifetime days RR = 3.29; 95% CI, 1.40–7.73; *p*-trend = 0.02; intensity-weighted lifetime days RR = 2.57; 95% CI, 1.28–5.17; *p*-trend = 0.002). This trend was not observed when the referent group comprised applicators who never used dicamba. There were no differences in risk for lung or colon cancer when we restricted the analysis to applicators who first applied dicamba prior to 1990 (data not shown). NHL was not associated with dicamba exposure, and there were too few cases of multiple myeloma to analyze.

We attempted to stratify risk for all cancers by state of residence (Iowa vs. North Carolina) and applicator type (private vs. commercial) but were unable to provide stable estimates for most cancers in North Carolina because of small numbers. For example, there were only 6 dicamba-exposed colon cancer cases in North Carolina, and 12 dicamba-exposed lung cancer cases. Given that a large proportion of participants with no dicamba exposure came from North Carolina (50%) and the majority of our highly exposed participants came from Iowa (92%), state of residence is clearly associated with dicamba exposure. The trends in risk for lung and colon cancer did not significantly change after restricting the analyses to the state of Iowa.

We also examined risk for each cancer site for each of the three components of the exposure algorithm grouped into tertiles: days per year of dicamba use, total years of dicamba use, and the dicamba intensity score. We observed no association between any of these components and risk for any cancer site, except for lung cancer, where the RR was 2.07 (95% CI, 1.05–4.08) for participants in the top tertile (> 15.5 years of dicamba exposure; *n* = 13 exposed cases) relative to that for unexposed participants (data not shown). Because dicamba is often used in combination with the herbicides 2,4-D, atrazine, or glyphosate, we attempted to examine dicamba-associated risk of lung and colon cancers stratified by never/ ever use of these three herbicides. However, a large proportion of applicators reported ever using 2,4-D, atrazine, and glyphosate (78, 72, and 77%, respectively), so the numbers of dicamba-exposed cases in the never-used strata were too small to provide a meaningful comparison of risk between strata.

Last, we attempted to adjust for confounding due to concurrent exposure to other pesticide in multiple ways. We examined the effect of adjusting for ever/never use of nine other pesticides most highly correlated with dicamba use. None of the individual pesticides substantially altered the trends in risk. When all nine pesticide variables were entered into the models simultaneously, the magnitude of adjustment was similar to when we used total lifetime days of any pesticide use in continuous form. For the sake of efficiency we selected total lifetime days of any pesticide use as the main adjustment variable. We also subtracted the number of dicamba lifetime exposure days from the total lifetime days of any pesticide use. This did not substantially alter the trends in risk for any of the cancer sites we examined; almost all point estimates remained the same.

## Discussion

Our study is the largest study to date of cancer risk associated with exposure to the herbicide dicamba. We observed a suggestion of increased risk for lung and colon cancer when the referent group comprised low-exposed applicators but not when the referent group comprised unexposed applicators. With further follow-up and accumulation of exposed cases, we believe we will better understand this phenomenon.

The association with lung cancer we observed in our study is similar to that reported in an earlier nested case–control study in the AHS cohort. Alavanja et al. (2004) observed a positive trend in risk for lung cancer with lifetime exposure days of dicamba, for the highest exposure tertile (highest tertile divided at the median) relative to the lowest exposure tertile [odds ratio (OR) = 1.0, 1.3, 1.7, 3.1; *p*-value for trend = 0.04). A significant trend was not observed when the investigators used unexposed participants as the referent group (OR = 1.0, 0.7, 0.9, 1.1, 1.6; *p*-value for trend = 0.15). When we modeled risk of lung cancer associated with lifetime dicamba exposure days using the low exposed as the referent group and the same tertile cut points used in the analysis conducted by Alavanja et al. (2004), not surprisingly, our results were very similar (OR = 1.0, 1.1, 1.6, 2.1; *p*-value for trend = 0.02). There is no prior evidence that suggests an association between dicamba exposure and colon cancer.

Exposure to dicamba has been associated with increased risk for NHL in a few previous case–control studies. In a case–control study of NHL and pesticide exposure conducted in Canada, information on pesticide exposure was collected through a combination of mailed questionnaires and telephone interviews (McDuffie et al. 2001). After adjusting for demographic characteristics, family history of cancer in a first-degree relative, and history of selected medical conditions, increasing days per year of dicamba application was associated with an increased risk of NHL (OR = 1.7; 95% CI, 1.0–2.8). When exposure to dicamba as a general class was evaluated, which included dicamba-only products as well as mixtures of dicamba and glyphosate and mixtures of dicamba, 2,4-D and mecoprop, NHL risk increased slightly with increasing days per year of application (OR = 1.9; 95 %CI, 1.3–2.7). Conversely, results from a case–control study of NHL and farming in the United States suggested no association between risk of NHL and ever handling either benzoic acids as a class (OR = 1.3; 95% CI, 0.9–1.9) or dicamba in particular (OR = 1.2; 95% CI, 0.7–2.0) (Cantor et al. 1992). After restricting the analyses to pesticides handled prior to 1965, risk for NHL was elevated among dicamba users (OR = 2.8; 95% CI, 0.96–8.1). Our prospective data do not provide evidence of an association between dicamba and NHL. Our findings, however, may be influenced by the small number of cases and relatively short follow-up time.

Results from other studies provide no evidence for an association between dicamba and risk of leukemia (OR = 0.7; 95% CI, 0.4–1.4) (Brown et al. 1990) or multiple myeloma (OR = 1.3; 95% CI, 0.6–2.8) (Brown et al. 1993). However, Burmeister (1990) reported a nonsignificant, marginal association between exposure to benzoic acids as a class and risk of multiple myeloma (OR = 1.22; confidence interval/*p*-value not reported). In our study, exposure to dicamba is likely to be a combination of exposure to dicamba-only products as well as to dicamba mixtures, making it difficult to disentangle the effect of dicamba from other pesticides included in dicamba mixtures. After stratifying dicamba models for lung and colon cancers by never/ever use of 2,4-D, atrazine, and glyphosate (three herbicides commonly mixed with dicamba), there was no evidence for either increased risk among dicamba-only users, or for increased risk among participants who also used 2,4-D, atrazine, or glyphosate. Because this analysis is based on information pertaining to ever/never use of individual pesticide active ingredients, however, we were unable to unambiguously differentiate between use of dicamba-only products and dicamba mixtures at this time.

There is little experimental evidence to suggest that dicamba is carcinogenic or mutagenic (U.S. EPA 1999). Feeding studies in rats, mice, dogs, and rabbits have shown no increased incidence of tumors (Extension Toxicology Network 1999). There is evidence that dicamba acts as a peroxisome proliferator (PP) by increasing fatty acyl–coenzyme A oxidase activity in the livers of rats and activating the peroxisome proliferator receptor in a dose-dependent fashion (Espandiari et al. 1998, 1999). It is thought that PPs may induce liver tumors in rats through mechanisms related to oxidative stress, inducing replicative DNA synthesis, or by promoting growth of preneoplastic lesions (Espandiari et al. 1998). Dicamba induced DNA damage in one study of rats (Perocco et al. 1990). In another study of mice, dicamba caused mortality in two of four mice injected with dicamba but did not increase xenobiotic-metabolizing activities in the two surviving mice (Moody et al. 1991). Few epidemiologic studies on the effect of PPs on humans have been conducted (Nakajima et al. 2002), but there are marked species differences in response to PPs (Lai 2004). Humans seem to exhibit a weak response to PP chemicals (including certain pesticides, industrial solvents, and hypolipidemic drugs), which may be due to low levels of peroxisome proliferator-activated receptor alpha in human liver (Lai 2004; Maloney and Waxman 1999).

The Agricultural Health Study is the largest study to date of pesticide applicators exposed to dicamba. The potential for recall bias is minimal, as exposure information was collected prior to cancer diagnosis. AHS applicators have been shown to provide reliable information about their histories of pesticide use (Blair et al. 2002; Hoppin et al. 2002), although misclassification can occur. Misclassification in a prospective study is likely to be nondifferential with regard to cancer occurrence and, although it could diminish estimates of relative risk, it is unlikely to create false positives (Checkoway et. al. 2004).

Our results may be affected by simultaneous exposure to other pesticides of varying intensity that has changed over time, although we attempted to account for this by adjusting for total lifetime days of any pesticide use. In addition, we could not differentiate use of dicamba-only products from dicamba mixtures. This is one of the biggest challenges in conducting epidemiologic research on pesticides, as many pesticides are most often used in combination with others in complex mixtures and not as individual pesticides. The existing toxicologic data, however, pertain to dicamba as an individual chemical. Our findings may also be limited because of a relatively short period of follow-up and small numbers of cases for some cancer sites. Because of the lack of consistency among results from the four evaluations of exposure metric and referent type for lung and colon cancer, these findings should be interpreted with caution.

Despite these limitations, our prospective study of cancer incidence among dicamba-exposed pesticide applicators provided an opportunity afforded in few other studies to evaluate cancer risks associated with exposure to dicamba while adjusting for lifetime use of other pesticides and lifestyle factors. We did not detect much evidence for an association between dicamba exposure and any of the cancer sites investigated, but the patterns of associations observed for lung and colon cancers warrant further attention. We will re-examine dicamba in the future when larger numbers will allow for a more comprehensive evaluation of lung and colon cancer, as well as additional cancer sites.

## Figures and Tables

**Table 1:** Selected characteristics of male applicators enrolled in the Agricultural Health Study categorized by dicamba exposure, 1993–2001 [no. (%)].

Characteristic	Unexposed (*n* = 19,933)	Low-exposed^a^ (*n =* 6,116)	High-exposed^b^ (*n =* 15,920)
Age (years)			
< 40	7,325 (36.7)	2,059 (33.7)	5,190 (32.6)
40–49	5,234 (26.3)	1,838 (30.0)	5,269 (33.1)
50–59	3,855 (19.3)	1,235 (20.2)	3,349 (21.0)
≥ 60	3,519 (17.7)	984 (16.1)	2,112 (13.3)
Race			
White	19,362 (97.1)	6,094 (99.6)	15,850 (99.6)
Nonwhite	571 (2.9)	22 (0.4)	70 (0.4)
State			
Iowa	9,911 (49.7)	5,350 (87.5)	14,693 (92.3)
North Carolina	10,022 (50.3)	766 (12.5)	1,227 (7.7)
Smoking			
Never	10,359 (52.0)	3,736 (61.2)	9,153 (57.5)
Former	4,710 (23.6)	1,378 (22.5)	3,716 (23.3)
Current	4,864 (24.4)	1,002 (16.4)	3,051 (19.2)
Education			
≤ High School	11,005 (55.2)	3,042 (49.7)	8,175 (51.3)
> High School	8,490 (46.2)	2,983 (48.8)	7,541 (47.4)
Missing	438 (2.2)	91 (1.5)	204 (1.3)
Alcohol consumption^c^			
No	7,518 (37.7)	1,521 (24.9)	3,093 (19.4)
Yes	12,415 (62.3)	4,595 (75.1)	12,827 (80.6)
Family history, cancer			
No	12,332 (61.9)	3,510 (57.4)	9,201 (57.8)
Yes	7,601 (38.1)	2,606 (42.6)	6,719 (42.2)
Applicator type			
Private	11,822 (59.3)	5,277 (86.3)	13,679 (85.9)
Commercial	8,111 (40.7)	839 (13.7)	2,241 (14.1)
Own or work on farm^c^			
Never	2251 (11.3)	275 (4.5)	1,281 (8.0)
Ever	17,568 (88.1)	5834 (95.4)	1,4615 (91.8)
Missing	114 (0.6)	7 (0.1)	24 (0.2)
Field corn production			
No	8,111 (40.7)	839 (13.7)	2,241 (14.1)
Yes	11,822 (59.3)	5,277 (86.3)	13,679 (85.9)
Soybean production			
No	8,403 (42.2)	1,324 (21.6)	3,155 (19.8)
Yes	11,530 (57.8)	4,792 (78.4)	12,765 (80.2)
Person-years (total)	148,314.7	44,557.4	115,806.4
Follow-up (years)^d^	7.4 ± 1.5	7.3 ± 1.4	7.3 ± 1.4
Total lifetime days of pesticide application^d^	345.0 ± 617.2	278.0 ± 434.9	472.9 ± 615.6

a First tertile of lifetime exposure-days (years of use × days per year).

b Second and third tertiles of lifetime exposure days.

c During enrollment year.

d Mean ± SD.

**Table 2:** Rate ratios and 95% CI for selected cancers by tertiles of total dicamba lifetime exposure days among male pesticide applicators in the Agricultural Health Study.^a^

		Dicamba exposure			
		No exposure—referent		Low exposed—referent	
Cancer site	Cases (*n*)	RR	95% CI	RR	95% CI
All cancers					
No exposure	887	1.00			
1 to < 20	227	0.90	0.77–1.05	1.00	
20 to < 56	254	1.00	0.82–1.11	1.07	0.89–1.28
56 to < 116	169	0.90	0.73–1.03	0.97	0.79–1.19
≥ 116	157	1.02	0.85–1.23	1.18	0.94–1.46
			*p* = 0.69		*p* = 0.18
Colon					
No exposure	76	1.00			
1 to < 20	9	0.42	0.20–0.85	1.00	
20 to < 56	20	0.88	0.52–1.50	2.07	0.94–4.57
56 to < 116	13	0.81	0.43–1.51	1.85	0.79–4.37
≥ 116	17	1.42	0.78–2.58	3.29	1.40–7.73
			*p* = 0.10		*p* = 0.02
Lung					
No exposure	95	1.00			
1 to < 20	14	0.84	0.45–1.54	1.00	
20 to < 56	11	0.64	0.33–1.26	0.82	0.36–1.85
56 to < 116	12	0.96	0.50–1.85	1.29	0.58–2.89
≥ 116	15	1.47	0.79–2.72	2.16	0.97–4.82
			*p* = 0.13		*p* = 0.02
Prostate					
No exposure	343	1.00			
1 to < 20	106	1.00	0.80–1.27	1.00	
20 to < 56	102	0.94	0.74–1.20	0.94	0.72–1.24
56 to < 116	76	0.96	0.73–1.25	0.95	0.71–1.29
≥ 116	67	1.08	0.81–1.46	1.10	0.79–1.53
			*p* = 0.45		*p* = 0.45
Bladder					
No exposure	43	1.00			
1 to < 20	6	0.51	0.21–1.25	1.00	
20 to < 56	9	0.66	0.31–1.43	1.26	0.44–3.55
56 to < 116	6	0.59	0.24–1.45	1.11	0.36–3.47
≥ 116	8	0.82	0.36–1.88	1.39	0.44–4.42
			*p* = 0.66		*p* = 0.66
Melanoma					
No exposure	32	1.00			
1 to < 20	10	0.97	0.46–2.06	1.00	
20 to < 56	18	1.59	0.84–3.00	1.65	0.76–3.60
56 to < 116	6	0.72	0.29–1.81	0.75	0.27–2.07
≥ 116	6	0.83	0.33–2.13	0.93	0.32–2.71
			*p* = 0.51		*p* = 0.48
Non-Hodgkin lymphoma					
No exposure	39	1.00			
1 to < 20	18	1.75	0.96–3.21	1.00	
20 to < 56	14	1.29	0.66–2.53	0.73	0.36–1.48
56 to < 116	7	0.92	0.39–2.16	0.54	0.22–1.31
≥ 116	7	1.19	0.50–2.85	0.76	0.30–1.97
			*p* = 0.92		*p* = 0.71
All hematopoietic^b^					
No exposure	82	1.00			
1 to < 20	31	1.38	0.89–2.15	1.00	
20 to < 56	32	1.37	0.87–2.14	0.97	0.59–1.59
56 to < 116	16	0.96	0.54–1.70	0.69	0.37–1.26
≥ 116	17	1.31	0.74–2.31	0.99	0.53–1.87
			*p* = 0.66		*p* = 0.99

a Adjusted for age, state of residence, smoking (pack-years), education, family history of cancer, and total lifetime days of pesticide application; upper tertile divided at the median.

b Includes leukemia, multiple myeloma, Hodgkin lymphoma, and non-Hodgkin lymphoma.

**Table 3:** Rate ratios and 95% CI for selected cancers by tertiles of dicamba intensity-weighted lifetime exposure days among male pesticide applicators in the Agricultural Health Study.^a^

		Dicamba exposure			
		No exposure—referent		Low exposed—referent	
Cancer site	Cases (*n*)	RR	95% CI	RR	95% CI
All cancers					
No exposure	888	1.00			
1 to < 86.6	251	0.90	0.80–1.00	1.00	
86.6 to < 344.25	278	1.00	0.84–1.13	1.11	0.93–1.32
344.25 to < 739.2	131	0.90	0.73–1.08	1.02	0.82–1.26
≥ 739.2	144	1.00	0.82–1.20	1.15	0.93–1.43
			*p* = 0.91		*p* = 0.35
Colon					
No exposure	76	1.00			
1 to < 86.6	16	0.64	0.36–1.14	1.00	
86.6 to < 344.25	17	0.70	0.40–1.22	1.04	0.52–2.06
344.25 to < 739.2	6	0.50	0.21–1.17	0.74	0.29–1.91
≥ 739.2	20	1.76	1.00–3.07	2.57	1.28–5.17
			*p* = 0.02		*p* = 0.002
Lung					
No exposure	95	1.00			
1 to < 86.6	11	0.61	0.31–1.21	1.00	
86.6 to < 344.25	20	1.07	0.62–1.84	1.92	0.89–4.11
344.25 to < 739.2	10	1.03	0.51–2.08	1.90	0.78–4.60
≥ 739.2	11	1.10	0.56–2.18	2.20	0.90–5.38
			*p* = 0.58		*p* = 0.21
Prostate					
No exposure	343	1.00			
1 to < 86.6	115	0.97	0.77–1.21	1.00	
86.6 to < 344.25	110	0.95	0.75–1.20	1.00	0.75–1.27
344.25 to < 739.2	59	1.03	0.77–1.37	1.07	0.78–1.46
≥ 739.2	61	1.11	0.83–1.50	1.17	0.84–1.62
			*p* = 0.38		*p* = 0.27
Bladder					
No exposure	43	1.00			
1 to < 86.6	6	0.46	0.19–1.11	1.00	
86.6 to < 344.25	13	0.89	0.45–1.74	1.95	0.74–5.13
344.25 to < 739.2	6	0.77	0.31–1.90	1.70	1.54–5.27
≥ 739.2	4	0.43	0.15–1.25	0.94	0.26–3.41
			*p* = 0.20		*p* = 0.34
Melanoma					
No exposure	32	1.00			
1 to < 86.6	10	0.84	0.40–1.80	1.00	
86.6 to < 344.25	18	1.51	0.80–2.84	1.80	0.83–3.91
344.25 to < 739.2	7	1.06	0.45–2.53	1.27	0.48–3.35
≥ 739.2	5	0.77	0.28–2.07	1.00	0.32–2.90
			*p* = 0.60		*p* = 0.54
Non-Hodgkin lymphoma					
No exposure	39	1.00			
1 to < 86.6	17	1.43	0.77–2.67	1.00	
86.6 to < 344.25	18	1.55	0.83–2.87	1.08	0.56–2.10
344.25 to < 739.2	4	0.67	0.23–1.91	0.46	0.15–1.37
≥ 739.2	6	1.07	0.43–2.67	0.74	0.29–1.93
			*p* = 0.68		*p* = 0.51
All hematopoietic^b^					
No exposure	82	1.00			
1 to < 86.6	31	1.22	0.78–1.90	1.00	
86.6 to < 344.25	35	1.41	0.91–2.18	1.13	0.70–1.84
344.25 to < 739.2	16	1.23	0.69–2.17	1.00	0.54–1.82
≥ 739.2	12	1.00	0.51–1.86	0.83	0.41–1.66
			*p* = 0.81		*p* = 0.46

a Adjusted for age, state of residence, smoking (pack-years), education, family history of cancer, and total lifetime days of pesticide application; upper tertile divided at the median.

b Includes leukemia, multiple myeloma, Hodgkin lymphoma, and non-Hodgkin lymphoma.
